# St. Katherine's Hospital

**Published:** 1888-07-07

**Authors:** 


### St. Katherine's Hospital.

From a Correspondent.

As there now appears to be some prospect of this ancient and wealthy foundation being applied to a use more in accordance with the intentions of its founders than it has been of late years, a few notes on its early history may be of interest.

It was originally founded, in 1148, by Matilda of Boulogne, wife of King Stephen, for the maintenance of a master and several poor brethren and sisters. The hospital was on the east of the Tower of London, and there remained until 1825, when it was removed to make way for the St. Katherine's Docks, and rebuilt in Regent's Park. The old foundation was dissolved in 1273 by Eleanor, widow of Henry III., who reconstituted it for a master and three brethren (who were all to be priests), three sisters, ten bedeswomen, and six poor scholars. These were all to be devoted to works of charity and almsgiving among the poor, as examples of Christian life and character, and must have possessed much influence over the numerous sailors of all nations with whom they came in contact. The brethren were to wear "a strait coat," and over that a black mantle, with "the sign of the holy Katherine," the well-known Katherine wheel, now chiefly associated with fireworks, but originally intended to keep in remembrance the manner of Saint Katherine's death. Coloured clothes were not to be worn. Pope Urban IV., who was opposed to this alteration in the statutes, endeavoured in vain to reinstate the former prior and brotherhood, who had plundered the hospital and neglected their duties. St. Katherine's, in fact, seems always to have suffered from the rapacity and mismanagement of those who should have protected its interests.

Philippa of Hainault, wife of Edward III., founded a chantry, and gave houses in Kent and Herts, besides £10 per annum in land, for an additional chaplain. Henry VI. was likewise a benefactor, but the chief one was Thomas de Bekington, afterwards Bishop of Bath and Wells. He, being master in 1445, obtained a charter by which the precincts of the hospital were declared free from all external jurisdiction, ecclesiastical or civil, except that of the Lord Chancellor. An annual fair of twenty-one days, commencing on St. James's Day (July 25), was to be held on Tower Hill. Henry VIII. and Katharine of Arragon founded here the Guild of St. Barbara, governed by a master and three wardens. Cardinal Wolsey and most of the chief nobility were members. King Henry confirmed the liberties and franchise of this house in 1527, and by some means, probably at the intercession of Anne Boleyn, it escaped dissolution in 1534. This fate, however, befel it in the next reign, when it was seized by the king.

In the reign of Elizabeth, her secretary, Thomas Wilson, although a layman, was made master, in direct contravention of the statutes. He forthwith commenced plundering the hospital by surrendering the old charter of Henry VI., and obtaining a new one, in which all mention of the great annual fair was omitted. He then sold the rights of the fair for £466 13*s.* 4*d.* to the Corporation of London, and endeavoured to seize upon all the other estates belonging to the hospital, but his lawless proceedings caused such an out cry that even Secretary Cecil was compelled to interfere.

In 1672 an enemy of another kind appeared. A dreadful fire destroyed 100 houses in the precincts, which are described by Stow as holding more inhabitants than some cities of England; and in 1734 thirty more were burnt. The church, as having been built in Popish times, was in some slight danger during the Gordon riots in 1780, but a display of force saved it. This church was an interesting building in the Perpendicular style, and contained some tombs, which are now in the church at Regent's Park. One of these is the tomb of John Holland, Duke of Exeter and Constable of the Tower, who died in 1447. It is an altar tomb, and has the effigies of the duke and his two wives under a rich canopy.

The Queen Consorts of England are by law the perpetual patronesses of the hospital, which forms the only piece of preferment in the gift of the Queen Consort for the time being. If, however, there is a Queen Dowager, she retains the right during her life.

